# Sofosbuvir: A Potential Treatment for Ebola

**DOI:** 10.3389/fphar.2018.01139

**Published:** 2018-10-10

**Authors:** Sandra E. Reznik, Amit K. Tiwari, Charles R. Ashby

**Affiliations:** ^1^Department of Pharmaceutical Sciences, College of Pharmacy and Health Sciences, St. John's University, Queens, NY, United States; ^2^Departments of Pathology and Obstetrics and Gynecology and Women's Health, Montefiore Medical Center/Albert Einstein College of Medicine, Bronx, NY, United States; ^3^Department of Pharmacology and Experimental Therapeutics, College of Pharmacy and Pharmaceutical Sciences, University of Toledo, Toledo, OH, United States

**Keywords:** Ebola, sofosbuvir, RNA virus, RNA dependent RNA polymerase, triphosphate metabolite

There are currently no effective licensed vaccines or proven drugs available for the treatment of Ebola, which causes one of the deadliest viral diseases. The urgent need to identify novel and effective pharmacologic approaches to treat Ebola infections is underlined by recent reports of new Ebola outbreaks from the Centers for Disease Control and Prevention (CDC), including the outbreak in the Eastern Democratic Republic of the Congo on August 1, 2018. Here we present an argument as to why sofosbuvir, presently used to treat chronic hepatitis C virus (HCV) infections, could be an ideal candidate for the treatment of Ebola infection.

Ebola is a negative-sensed, non-segmented RNA virus and as with all RNA viruses, including HCV, uses the enzyme RNA-dependent RNA polymerase (RdRp, transcribed from the Ebola L gene), along with other proteins, to replicate, maintain, and express its RNA genome (Trunschke et al., 2013) by (1) binding to an appropriate complementary 5′-nucleotide triphosphate and converting it to a 5′-nucleotide monophosphate and (2) catalyzing the interaction between the NMP and a 3′-hydroxyl-ribonucleotide to form a 5′-3′ phosphodiester bond (McDonald, 2013). This process elongates the daughter RNA strand. The three dimensional structure of RdRp from RNA viruses resembles that of a cupped right hand and contains a finger, thumb, and palm domain (McDonald, 2013). Further, the palm domain of all viral RdRps, which mediate catalysis for RNA polymerization, have motifs A-E, and motifs A-C are the most conserved (Jácome et al., 2015). The predicted three-dimensional structure of the Ebola RdRp is similar to other viral RdRp (including HCV) as it has the homologous and highly conserved catalytic domains A-C in the palm (Jácome et al., 2015). Furthermore, like all other viral RdRp, Ebola RdRp utilizes 2 divalent metal ions to catalyze ribonucleotide polymerization (Jácome et al., 2015). The Ebola RdRp is an excellent pharmacological target as (1) its inhibition will decrease viral replication; (2) there is no similar protein target in human cells.

Sofosbuvir (400 mg p.o. once daily for 12 or 24 weeks), in combination with other drugs, is used to treat chronic HCV infections (Keating, 2014). Sofosbuvir is a uridine analog nucleotide phosphoramidate prodrug that is ultimately bio-transformed in hepatocytes to a triphosphate metabolite (GS-461203) that inhibits HCV RdRp (also known as the NS5B protein)-catalyzed RNA synthesis, thereby inhibiting viral replication and transcription (Sofia, 2013). GS-461203 has a half-life of 38 h in human hepatocytes (Summers et al., 2014), and reaches concentrations that exceed the EC_50_ for Ebola RdRp, ensuring that it will produce sustained antiviral action. Interestingly, the triphosphate metabolite of the adenine analog BCX4430 inhibits HCV RdRp *in vitro* and protects mice against Ebola (Warren et al., 2014). Furthermore, the triphosphate metabolite of the nucleoside analog favipiravir (T-705) inhibits RdRp activity of numerous RNA viruses and protects mice against Ebola-induced mortality 6 days after infection (Oestereich et al., 2014). However, there was no statistically significant decrease in the mortality rate in patients from Guinea (Sissoko et al., 2016) and Sierra Leone (Bai et al., 2016) during the 2013–2016 Ebola epidemic patients that received favipiravir. Furthermore, there were patients who were evacuated from West Africa to Europe who received favipiravir, but its effect on patient survival could not be determined as all of these patients received advanced supportive care and the majority were given additional experimental treatment (Mora-Rillo et al., 2015; Schibler et al., 2015; Agrati et al., 2016). It should be noted that in the JIKI trial (single-arm, proof-of-concept design), the plasma concentrations of favipiravir did not reach the target levels set prior to the trial (Nguyen et al., 2017).

The i.v. administration of 10 mg/kg of GS-5734 (once daily for 12 days), an adenine analog nucleotide phosphoramidate prodrug that is bio-transformed to an active nucleoside triphosphate compound, significantly decreased Ebola virus replication and protected 100% of rhesus monkeys against lethal Ebola virus (from 1995 outbreak in Kikwit, Zaire) infection (Warren et al., 2016). GS-5734 inhibits (EC_50_ = 86 nM) Ebola virus replication in human macrophages (Siegel et al., 2017). Furthermore, GS-5734 has completed phase I trials and is undergoing Phase II trials to determine its efficacy in Ebola survivors who have persistent viremia in semen (Bixler et al., 2017). The triphosphate metabolites of sofosbuvir (Sofia, 2013), BCX4430 (Warren et al., 2014), favipiravir (Bai et al., 2016), and GS-5734 (Warren et al., 2016) are incorporated into the viral RNA template and inhibit RdRp activity via chain termination. Thus, certain compounds that are active against HCV RdRp may also be efficacious in inhibiting Ebola RdRp.

We propose that prior to clinical trials, sofosbuvir's efficacy be tested *in vitro* against human macrophages and Huh-7 cells infected with the Ebola Makona variant. If these results indicate that sofosbuvir is efficacious, we propose that its *in vivo* efficacy be determined in a non-human primate model of Ebola. If these results are positive, sofosbuvir's efficacy in humans could be determined by measuring Ebola virus RNA in the semen of males 18–65 years old (*n* = 40) who were identified as having had a PCR-confirmed Ebola diagnosis in a double-blind, randomized, placebo-controlled trial. A 400-mg dose of sofosbuvir would be administered orally once daily for 28 days to individuals in the test group and a placebo tablet given to the control participants. The presence of Ebola RNA in all samples would be determined using real-time RT-PCR. Samples would be obtained once per week during treatment and once per month thereafter. The presence of infectious Ebola virus in the semen samples would be determined in severe combined immunodeficient mice as previously described (Sissoko et al., 2017). During treatment, all patients would be monitored for potential adverse effects via interviews and by obtaining blood samples. Following the treatment period, samples would be collected once a month for at least 13 months. All collected seminal fluid samples in the trial proposed here would be tested for the presence of Ebola virus RNA, and the clearance rate of the virus over 13 months in the two groups would be compared. If there is a statistically significant increase in viral clearance in the treated group as compared to the placebo control group, the clinical efficacy of sofosbuvir in humans could be determined by identifying individuals 18 years of age or older in a future epidemic who have laboratory-confirmed Ebola infection for a double-blind, randomized, placebo-controlled trial. Recent data suggest that the mortality rate from Ebola is about 50%. Using this conservative estimate, and the modest prediction that sofosbuvir would decrease the mortality rate to 30%, sample sizes of *n* = 60 each for the control and drug groups would be sufficient to detect a statistically significant effect (*p* < 0.05). A 400-mg dose of sofosbuvir would be administered orally once daily for 28 days to individuals in the test group and a placebo tablet given to the control participants. The main efficacy endpoint would be survival after 28 days. Also, all placebo and sofosbuvir patients would receive the best available supportive care. Since we do not know whether sofosbuvir will significantly lower the mortality rate, the use of placebo is justified. Finally, patients should be followed to determine their viral load and acquired resistance to sofosbuvir.

Clinical studies and post-marketing data suggest that sofosbuvir has a highly favorable safety profile (Keating and Vaidya, 2014). The most common adverse effects produced by sofosbuvir are headache, nausea, dizziness, fatigue and abdominal pain, and no dose-limiting toxicities have been reported (Keating and Vaidya, 2014). Nonetheless, sofosbuvir treatment will be discontinued if it elicits severe or problematic adverse effects or increases mortality.

Patients should be screened for HCV as using sofosbuvir alone for the treatment of HCV would not be considered optimal therapy. It is recommended that sofosbuvir should not be taken by women who are breastfeeding. Animal data indicate that sofosbuvir and its predominant circulating metabolite, GS331007, at doses far exceeding those used in HCV patients, does not produce carcinogenicity, mutagenicity, or an impairment of fertility (Product Information, Gilead Sciences, Inc., 2015). A recent *in vitro* study indicates that sofosbuvir, at high concentrations, does not have toxic effects on the following human cell lines: hepatic (Huh7, HepG2), prostate (PC-3), fibroblasts (MRC5), T cells (MT-4), bone marrow erythroid, and myeloid cells (Feng et al., 2016). Finally, sofosbuvir (>200 μM) does not significantly inhibit (1) the activity of the mitochondrial DNA polymerases alpha, beta, and gamma or (2) mitochondrial protein synthesis and respiration in PC-3 cells (Feng et al., 2016).

Clinical data suggest that sofosbuvir's pharmacokinetic profile is highly suitable for the potentially diverse populations of patients presenting with Ebola infection (Kirby et al., 2015). Notably, sofosbuvir has a large volume of distribution (127 l), thereby increasing the likelihood that sufficient concentrations will be present in reservoir areas (e.g., eyes, testes, CNS) that support active Ebola replication. Current guidelines indicate that sofosbuvir can be used in patients with severe liver impairment and mild or moderate [estimated glomerular filtration rate (eGFR) > 30 ml/min] impairment of renal function (Kirby et al., 2015). However, 4 recent studies (Hundemer et al., 2015; Dumortier et al., 2016; Nazario et al., 2016; Singh et al., 2016) in patients with chronic HCV (total of 80 patients) have reported that sofosbuvir (in combination with other anti-HCV drugs) for 12 to 24 weeks was well tolerated at doses of 400 mg/day, 400 mg every other day or 3 times a week in the presence of end stage renal disease or in patients with eGFR < 30 ml/min. Thus, the use of sofosbuvir could be considered in Ebola patients with severe renal impairment in the absence of alternatives and with careful monitoring. The absorption of sofosbuvir could be affected by vomiting or diarrhea and it cannot be given to patients who are unconscious or have the inability to swallow the tablets. These issues could be addressed by i.v. administration, but there is no i.v. formulation for sofosbuvir. Sofosbuvir could be given in a solution of sulfobutylether-beta-cyclodextrin, although the pharmacokinetic profile of this formulation remains to be determined. Alternatively, the sofosbuvir tablet could be disintegrated into water, juice, or milk with spoon stirring and light press and given via a nasoduodenal tube, although the pharmacokinetics of this formulation is unknown (Li and Foisy, 2014). Currently, the efficacy and safety of sofosbuvir for pediatric patients has not been reported.

Sofosbuvir and its main metabolite are not known to be substrates for CYP450 drug metabolizing enzymes and do not induce or inhibit these enzymes (Kirby et al., 2015). Sofosbuvir is a substrate for the ABC transporters p-glycoprotein (ABCB1) and breast cancer resistant protein (BRCP or ABCG2 transporter) (Kirby et al., 2015). Overall, sofosbuvir has a low liability to elicit significant drug-drug interactions. Indeed, the number of clinically significant drug-drug interactions are minimal for sofosbuvir (it should not be co-administered with potent inducers of intestinal ABCB1 and/or ABCG2, carbamazepine, oxcarbazepine, phenytoin, phenobarbital tipranivir + ritonavir, rifampin, rifabutin, rifapentine, or amiodarone) (Product Information, Gilead Sciences, Inc., 2015). This is important as Ebola patients may be receiving many drugs as part of their treatment regimen and based on published data, sofosbuvir is highly unlikely to attenuate the efficacy and/or increase the toxicity of numerous other drugs used to treat concomitant infections.

The cost of sofosbuvir is a critical issue regarding its use for Ebola. The median nominal ex-factory cost of a 12-week regimen of sofosbuvir for treating HCV, across 26 countries in the Organization for Economic Cooperation and Development (OECD), was $42,017 (Iyengar et al., 2016). This aforementioned price range would make sofosbuvir unavailable as a potential treatment to most patients on a global level. However, the cost of generic sofosbuvir in India, ranges from $161 to 312 for 28 tablets (Iyengar et al., 2016). Thus, a 4-week regimen of sosfobuvir, as proposed in this paper, would be projected to cost $161 to 312. Furthermore, it has been estimated that based upon (1) the manufacturing cost of retroviral drugs with similar mechanisms of action and chemical structures and (2) treating a minimum of 1 million people (Hill et al., 2014), a 12-week regimen of sofosbuvir should cost $68–136, or $23–45 for 4 weeks.

In conclusion, we hypothesize that sofosbuvir, a highly safe and effective treatment for HCV, if given in a timely manner, would decrease Ebola-induced mortality by lowering viral load. There is currently no drug that has proven to be efficacious against Ebola virus in a clinical setting, including favipiravir, and the safety profile of sofosbuvir is already well known. If sofosbuvir treatment significantly reduces Ebola mortality, its efficacy should be tested for prophylaxis and for post exposure prophylaxis. Also, its use could be considered in patients diagnosed with post-Ebola syndrome, given that one of the potential causes could be viral reservoirs.

## Author contributions

SR, CA, and AT discussed and agreed on the presented opinion. CA wrote the draft and SR and AT contributed to a portion of the draft. CA, AT, and SR proofed the article.

### Conflict of interest statement

The authors declare that the research was conducted in the absence of any commercial or financial relationships that could be construed as a potential conflict of interest.
